# Yeast Gdt1 is a Golgi-localized calcium transporter required for stress-induced calcium signaling and protein glycosylation

**DOI:** 10.1038/srep24282

**Published:** 2016-04-14

**Authors:** Anne-Sophie Colinet, Palanivelu Sengottaiyan, Antoine Deschamps, Marie-Lise Colsoul, Louise Thines, Didier Demaegd, Marie-Clémence Duchêne, François Foulquier, Pascal Hols, Pierre Morsomme

**Affiliations:** 1 Institut des Sciences de la Vie, Université catholique de Louvain, B-1348 Louvain-la-Neuve, Belgium; 2 UMR8576 CNRS, Structural and Functional Glycobiology Unit, University of Lille 1, IFR 114, F-59655 Villeneuve D’Ascq, France

## Abstract

Calcium signaling depends on a tightly regulated set of pumps, exchangers, and channels that are responsible for controlling calcium fluxes between the different subcellular compartments of the eukaryotic cell. We have recently reported that two members of the highly-conserved UPF0016 family, human TMEM165 and budding yeast Gdt1p, are functionally related and might form a new group of Golgi-localized cation/Ca^2+^ exchangers. Defects in the human protein TMEM165 are known to cause a subtype of Congenital Disorders of Glycosylation. Using an assay based on the heterologous expression of *GDT1* in the bacterium *Lactococcus lactis*, we demonstrated the calcium transport activity of Gdt1p. We observed a Ca^2+^ uptake activity in cells expressing *GDT1*, which was dependent on the external pH, indicating that Gdt1p may act as a Ca^2+^/H^+^ antiporter. In yeast, we found that Gdt1p controls cellular calcium stores and plays a major role in the calcium response induced by osmotic shock when the Golgi calcium pump, Pmr1p, is absent. Importantly, we also discovered that, in the presence of a high concentration of external calcium, Gdt1p is required for glycosylation of carboxypeptidase Y and the glucanosyltransferase Gas1p. Finally we showed that glycosylation process is restored by providing more Mn^2+^ to the cells.

As a closed compartment, the cell must adapt to environmental changes and has therefore developed intracellular signaling systems that can identify these stimuli and implement cellular responses to counteract the stress. In eukaryotic cells, calcium ions play a key role in the transduction of external signals into the cytosol. Upon stimulation, the signal is generated by a sudden, transient, and massive calcium influx into the cytosol from the external medium and/or internal stores. In the yeast *Saccharomyces cerevisiae*, large increases in the cytosolic calcium concentration have been observed in response to hypo- or hypertonic shock, sugar refeeding, mating pheromone α factor, or Ca^2+^-depletion of the secretory pathway^1^. Depending on the stress, calcium can either flow from the external medium through the low-affinity Ca^2+^ influx system^2^ and the high-affinity Ca^2+^ influx system (HACS), composed of three interacting proteins, Cch1p, Mid1p, and Ecm7^3^, or be released from the vacuole via the mechanosensitive Ca^2+^ channel Yvc1p^4^. The low basal cytosolic calcium concentration [in the range of 50 to 200 nM^5^] is then rapidly restored by specific transporters that actively pump Ca^2+^ out of the cytosol.

Accumulation of calcium in the cytosol is directly sensed by different Ca^2+^-binding proteins, of which calmodulin (CaM) is the best studied. The Ca^2+^-CaM complex activates several Ca^2+^-responsive signaling pathways, including the serine/threonine protein phosphatase calcineurin pathway. Calcineurin inhibits the vacuolar Ca^2+^/H^+^ exchanger Vcx1p through a, as yet, poorly understood post-translational regulatory mechanism^6^ and induces expression of *PMR1* and *PMC1* via dephosphorylation of the transcription factor Crz1p and its subsequent mobilization into the nucleus^7^. *PMR1* encodes a high affinity, low capacity, P-type Ca^2+^/Mn^2+^-ATPase primarily required for maintaining a suitable calcium concentration in the Golgi apparatus (around 200 μM) and, indirectly, in the endoplasmic reticulum (ER) (around 10 μM)^8^. Maintenance of an appropriate calcium concentration in secretory pathway organelles is essential for the activity of many Golgi- and ER-resident enzymes involved in the retention of luminal proteins, export of secretory proteins, and protein folding, degradation, and maturation^8^,^9^. Together with the vacuolar Ca^2+^-ATPase Pmc1p, Pmr1p also plays a crucial role in detoxifying the cytosol when high calcium concentrations are encountered in the environment, allowing the maintenance of low [Ca^2+^]_cyt_ levels^6^, and *pmr1* and *pmc1* mutants therefore show increased sensitivity to high external Ca^2+ ^10,11.

Although the calcium transport system has been intensively studied, the molecular identity of some transporters remains unknown. For instance, Miseta *et al*.^10^ suggested the existence of an unidentified Ca^2+^/H^+^ exchanger that, in the absence of *VCX1*, is activated upon Ca^2+^ stress . Other well-known examples are the putative transporters X and M responsible for the influx of external Ca^2+^ through the plasma membrane which have not yet been identified^12^.

We recently suggested that Gdt1p is a novel putative Golgi-localized Ca^2+^/cation antiporter in yeast^13^. Gdt1p belongs to the UPF0016 family, a highly conserved family of membrane proteins, the members of which display topological similarities with members of the cation/Ca^2+^ (CaCA) exchanger superfamily^14^,^15^. Like the Golgi Ca^2+^/Mn^2+^-ATPase Pmr1p, Gdt1p is involved in tolerance to high external Ca^2+^ concentrations. We previously showed that a strain lacking either of these transporters is sensitive to an increase in the external Ca^2+^ and that this sensitivity is increased by the loss of both transporters^13^, suggesting that Gdt1p and Pmr1p are involved in high Ca^2+^ stress tolerance by two distinct pathways and that one pathway can compensate for the absence of the other. Interestingly, the Ca^2+^ sensitivity of the *gdt1* mutant was subsequently shown to be suppressed by the expression of bacterial orthologs or the human ortholog TMEM165^13^,^15^, indicating conservation of function throughout evolution. A defect in the TMEM165 gene is known to cause a subtype of Congenital Disorder of Glycosylation (CDG), a group of rare diseases associated with impaired protein glycosylation^16^. At the cellular level, we have shown that TMEM165-deficient patients display acidification of the late endosomes and lysosomes and, using patch-clamp analysis in HeLa cells, have observed TMEM165-dependent cation transport^13^. Based on these data, we suggested that Gdt1p, TMEM165, and other members of the UPF0016 family could form a new group of Ca^2+^/cation antiporters regulating Ca^2+^ homeostasis^13^,^15^. The defects of glycosylation observed in TMEM165-deficient patients might be the result of an unbalanced Ca^2+^ concentration in organelles involved in the secretory pathway.

In this report, we present direct evidence that the budding yeast family member Gdt1p transports calcium. Using an *in vivo* transport assay in *Lactococcus lactis* cells expressing Gdt1p, we observed that Gdt1p promoted Ca^2+^ influx into the cytosol. Interestingly, Ca^2+^ influx was enhanced as the external pH increased, suggesting that Gdt1p couples calcium transport to proton transport and probably acts as a Ca^2+^/H^+^ antiporter. Furthermore, we showed in yeast that Gdt1p is involved in the Ca^2+^ response to environmental osmotic stress when Pmr1p, the major Ca^2+^ pump under normal conditions, is absent. The amplitude of the Ca^2+^ response was also found to increase with an increase in the cellular calcium stores. Importantly, we also showed that *GDT1* is required for glycosylation of carboxypeptidase Y and the glucanosyltransferase Gas1p, probably by maintaining an appropriate Ca^2+^ concentration in organelles involved in protein glycosylation. Strikingly we found that this defect was restored by the addition of Mn^2+^ in the external medium.

## Results

### Expression of yeast *GDT1* in *Lactococcus lactis*

**Figure 1:**
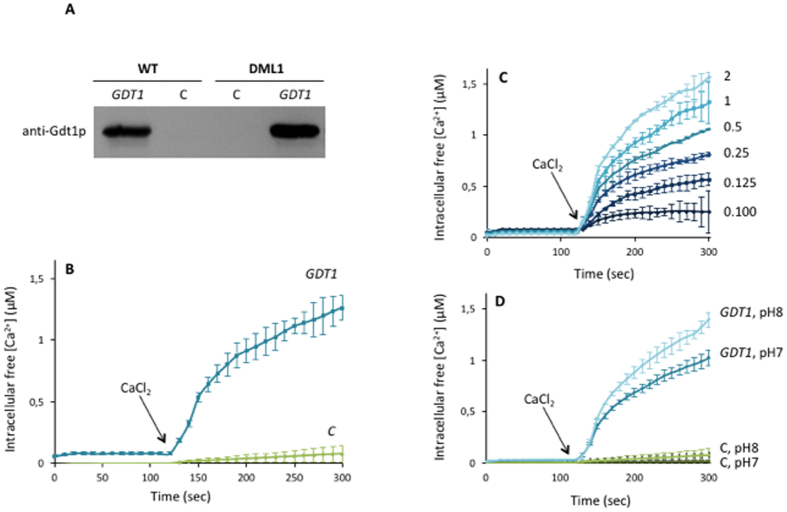
Gdt1p mediates calcium influx in *L. lactis* cells and this is strongly dependent on the external Ca^2+^ concentration and pH. (**A**) Wild type and evolved DML1 *L. lactis* cells expressing 10His-Strep-TEV-^Δ23^GDT1 were grown to an OD_600_ of 0.4–0.5 and Gdt1p expression was induced with nisin (2.5 μg/L). After 3 h of induction, the total membrane fraction was prepared and Gdt1p expression analyzed by SDS-PAGE followed by Western blotting with anti-Gdt1p antibodies. The negative control (**C**) consisted of the total membrane fraction from cells containing the empty pNZ8048 vector. (**B**) Calcium influx time course measurements performed in Fura-2-loaded DML1 cells expressing 10His-Strep-TEV-^Δ23^GDT1 or transformed with the empty vector pNZ8048 (**C**). After 3 h of induction, the cells were washed and resuspended in Ca^2+^-free assay medium pH 7.4. The fluorescence ratio (340/380) was recorded every 10 sec and converted into the [Ca^2+^]_cyt_ using the equation derived by Liao *et al*.^41^. The arrow indicates addition of 0.5 mM CaCl_2_. **(C)** Effect of the external Ca^2+^ concentration (right axis; mM) on Ca^2+^ accumulation in DML1 *L. lactis* cells expressing 10His-Strep-TEV-^Δ23^GDT1 at an extracellular pH of 7.4. **(D)** Effect of the external pH on Ca^2+^ accumulation in DML1 cells expressing 10His-Strep-TEV-^Δ23^GDT1 or transformed with the empty vector pNZ8048 (**C**) after addition of 0.5 mM CaCl_2_.

In order to investigate whether Gdt1p was involved in Ca^2+^ transport, we developed an *in vivo* functional transport assay based on the heterologous expression of Gdt1p in *L. lactis*, an organism that has been shown to be a valuable host for expressing eukaryotic membrane proteins^17^,^18^. The choice of this expression system was further supported by the absence of Gdt1p orthologs in *L. lactis*. As shown in Fig. 1A, Western blotting analysis showed that 10His-Strep-TEV-^Δ23^GDT1, a tagged version of Gdt1p lacking the first 23 amino acids predicted to be a signal peptide, could be expressed under the control of the nisin-inducible promoter in the wild type (WT) NZ9000 strain of *L. lactis*. We previously observed that the signal peptide of Gdt1p is not essential for the function of the protein and that this tagged version of Gdt1p is functional in yeast (data not shown). Nisin concentration and induction time were optimized to obtain the highest yield of Gdt1p (Fig. S1). Levels of expression were also compared in the WT strain and the “evolved” DML1 strain, which has been shown to be an efficient host for enhanced production of eukaryotic membrane proteins^19^. As shown in Fig. 1A, Gdt1p expression was clearly higher in the DML1 strain than in the WT, and DML1 was therefore chosen to set up the Ca^2+^ transport assay.

### Gdt1p promotes Ca^2+^ influx into *L. lactis* in a pH-dependent manner

To determine whether Gdt1p can function as a Ca^2+^ transporter, we used the Ca^2+^-sensitive fluorescent probe, Fura-2, to measure changes in the intracellular calcium concentration ([Ca^2+^]_cyt_) in *L. lactis* DML1 cells expressing *GDT1* or containing the empty vector. As shown in Fig. 1B, addition of 0.5 mM CaCl_2_ to DML1 cells expressing *GDT1* resulted in a marked increase in the [Ca^2+^]_cyt,_ whereas no increase was seen in control (C) cells lacking *GDT1*. Moreover, we observed that the [Ca^2+^]_cyt_ increased as the extracellular Ca^2+^ concentration was increased from 0.1 to 2 mM (Fig. 1C). Again, no increase was observed in control (C) cells lacking *GDT1* (data not shown). These results demonstrate that Gdt1p mediates Ca^2+^ transport across the plasma membrane when expressed in *L. lactis*. As mentioned above, Gdt1p shows striking similarities to members of the CaCA exchanger superfamily which transport Ca^2+^ across membranes against their electrochemical gradient by utilizing the downhill gradient of other cations, such as H^+^ or Na^+ ^14. Furthermore, mutation in the human ortholog TMEM165 has been shown to impair lysosomal and endosomal pH homeostasis^13^. For those reasons, we previously proposed that TMEM165 and Gdt1p might function as Ca^2+^/H^+^ antiporters, using the proton gradient as the driving force for Ca^2+^ influx across the Golgi membrane. To test this hypothesis, Fura-2-loaded *L. lactis* cells expressing tagged-^Δ23^Gdt1p were resuspended in assay buffer at different pH values (7.0 and 8.0) and Ca^2+^ accumulation was measured after addition of 0.5 mM CaCl_2_. As shown in Fig. 1D, Gdt1p clearly displayed Ca^2+^ influx activity that was dependent on the extracellular pH, with Ca^2+^ transport increasing as the external pH was increased from 7.0 to 8.0. This could be explained by the fact that proton extrusion from the cell is energetically more favorable at a higher pH and Gdt1p can use this energetically favorable condition to couple Ca^2+^ influx to the H^+^ efflux. No marked difference in the cell density of the Fura-2-loaded *L. lactis* cells was seen before and after CaCl_2_ addition, indicating that CaCl_2_ addition did not cause cell lysis, regardless of the external pH (Table S1). In addition, centrifugation of the cells after incubation with calcium and examining the fluorescence of the pellet and supernatant demonstrated that Fura-2 was not released into the extracellular medium, as less than 5% of the fluorescence was found in the supernatant and more than 95% in the pellet (data not shown). Together, these results demonstrate that Gdt1p mediates calcium influx in *L. lactis* and that this is regulated by the pH gradient, meaning that Gdt1p could be a Ca^2+^/H^+^ antiporter in yeast.

### Gdt1p is involved in calcium response to osmotic stress in yeast

Exposure of yeast cells to saline or osmotic stress triggers a sudden and transient increase in the [Ca^2+^]_cyt_ that results from Ca^2+^ influx through the plasma membrane channel Cch1p/Mid1p^20^ and release from the vacuole via the vacuolar channel Yvc1p^4^. The resting calcium level is then restored by reabsorption of calcium into the vacuole via the antiporter Vcx1p^4^.

**Figure 2:**
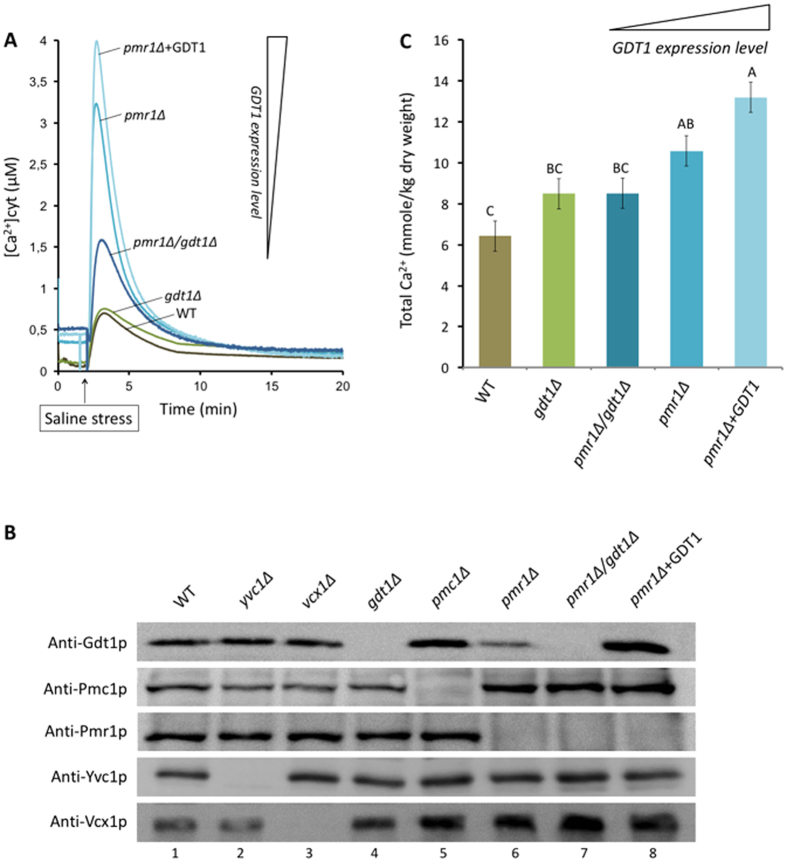
Gdt1p is involved in the calcium response to saline stress. **(A)** Wild type (WT) or various *pmr1* and/or *gdt1* yeast mutants expressing apo-aequorin from a plasmid were grown overnight to an OD_600_ of 1.2 in synthetic medium supplemented with coelenterazine (chromophore) to reconstitute the holoenzyme. Afterwards, 200 μL of each culture was transferred to luminometric tubes. After 2 min, NaCl (saline stress) was added at a final concentration of 1.33 M and the signal monitored for 20 min, then the lumimetric units were converted into the [Ca^2+^]_cyt_ using the equation from Allen *et al*.^42^. All displayed results are representative of those obtained in at least three replicates. **(B)** Proteins from the membrane-enriched fractions of exponentially growing cells (OD_600_ = 1.2) of the indicated strains were separated by SDS-PAGE and transferred to nitrocellulose membranes, which were then immunoblotted with antibodies against Gdt1p, Pmc1p, Pmr1p, Vcx1p, or Yvc1p. Coomassie blue-staining of the SDS-polyacrylamide gel indicated equal sample loading (Fig S3). **(C)** Cultures of the indicated strains were grown in synthetic medium to an OD_600_ of 3, then the cellular Ca^2+^ content was measured by ICP-AES on the dry matter. The data were analyzed by one-way analysis of variance (ANOVA) followed by a post-hoc Tukey-Kramer multiple comparisons test. The values are expressed as the mean ± S.E.M (n = 3). Letters not shared in two bars denote a significant difference (p < 0.05). The *pmr1*Δ* *+ GDT1 strain corresponds to the *pmr1*Δ mutant overexpressing *GDT1* under the control of the constitutive TPI1 promoter. Extracellular Ca^2+^ concentration for those three experiments was assessed by ICP-AES to be around 1 mM.

The role of Gdt1p in the calcium response following saline stress (1.33 M NaCl) was assessed in the WT and the *gdt1*Δ or *pmr1*Δ mutant by monitoring changes in the [Ca^2+^]_cyt_ using the genetically-encoded Ca^2+^ sensor aequorin. As shown in Fig. 2A, a similar calcium response to saline stress was observed on the WT and the *gdt1*Δ deletant; both strains had a low resting [Ca^2+^]_cyt_ of about 0.2 μM and the [Ca^2+^]_cyt_ increased sharply after exposure to stress, then returned to the basal level within 8 min. This shows that the loss of *GDT1* had no effect on the salt-induced calcium response. However, consistent with previous studies^3^,^21^, the basal [Ca^2+^]_cyt_ was markedly higher in the *pmr1*Δ mutant than in the WT (0.5 versus 0.2 μM). Loss of the Golgi Ca^2+^-ATPase Pmr1p is known to induce Ca^2+^-depletion in the secretory compartments that is compensated by a higher Cch1p/Mid1p-mediated Ca^2+^ influx and a net increase in the resting [Ca^2+^]_cyt_ is observed in cells grown under normal conditions^3^. As shown in Fig. 2A, addition of NaCl to *pmr1*Δ cells led to a higher calcium peak than in WT cells, suggesting either a higher rate of Ca^2+^ influx into the cytosol or a reduced reabsorption of Ca^2+^ into the organelles. We then tested whether Gdt1p was involved in the Ca^2+^ response in the absence of *PMR1* by monitoring the [Ca^2+^]_cyt_ in *pmr1*Δ cells lacking or overexpressing *GDT1*. The relative amount of Gdt1p detected in WT, *pmr1*Δ and *pmr1*Δ + GDT1 strains is shown in Fig. 2B. As previously reported, the level of Gdt1p is decreased in *pmr1*Δ cells compared to WT cells while overexpression of *GDT1* in *pmr1*Δ cells increases its level^13^. Interestingly, as shown in Fig. 2A, deletion of *GDT1* in *pmr1*Δ cells led to a smaller increase in the [Ca^2+^]_cyt_ than in *pmr1*Δ cells, while its overexpression (indicated as “ +GDT1”) resulted in a greater increase than in *pmr1*Δ cells. Similar calcium responses were observed after exposure of the cells to sorbitol (Fig. S2). Together, these results demonstrate that Gdt1p modulates the Ca^2+^ response in the *pmr1*Δ strain upon exposure of the cells to saline or osmotic stress. How does Gdt1p alter calcium responses? The simplest answer would be that it modulates the magnitude of the calcium peak by transporting Ca^2+^ from the Golgi to the cytosol. However, it was also possible that Gdt1p could act in an indirect manner, regulating levels of other Ca^2+^ transporters or altering internal Ca^2+^ stores and these possibilities were therefore examined.

### Gdt1p slightly modifies Vcx1p levels without affecting Pmr1p, Pmc1p, or Yvc1p levels

To assess the effect of Gdt1p on Pmr1p, Pmc1p, Yvc1p, and Vcx1p levels, we carried out Western blotting analysis on total membrane extracts from WT cells and cells expressing various mutants using specific antibodies. A specific signal was seen in the different strains analyzed, except in those strains in which the corresponding gene was deleted. As shown in Fig. 2B, Pmr1p and Yvc1p levels were similar in the different strains analyzed. In contrast, Pmc1p levels were higher in the *PMR1-*deleted strains (*pmr1*Δ, *pmr1*Δ/*gdt1*Δ, and *pmr1*Δ* + GDT1*). This result confirms the findings of Marchi *et al*.^22^, who reported calcineurin-dependent compensatory induction of the *PMC1* gene due to the loss of *PMR1*. This higher level of *PMC1* expression in *pmr1*Δ cells was unaffected by the level of expression of *GDT1* (lanes 6–8). Finally, Vcx1p levels were higher in the *gdt1*Δ cells (lane 4) than in the WT cells (lane 1) and were further increased in *pmc1*Δ cells (lane 5) and *pmr1*Δ cells (lane 6). Note that, in the *pmr1*Δ strain, Vcx1p levels were further increased after deletion of *GDT1* (lane 7) and decreased when *GDT1* was overexpressed (lane 8). These results rule out an indirect effect of Yvc1p, Pmr1p, and Pmc1p levels on the Gdt1p-dependent modulation of the Ca^2+^ response to osmotic stress, as their levels were not modified by deletion or overexpression of *GDT1*. In addition, the observed changes in Vcx1p levels cannot be linked to the Ca^2+^ responses, as, regardless of its level of expression in the *pmr1*Δ strains, Vcx1p should be inhibited as a result of calcineurin activation^6^. The activation of calcineurin in *PMR1-*deleted strains is suggested by the overexpression of PMC1 (Fig. 2B and Fig. S4B) and by the observation that steady-state [Ca^2+^]_cyt_ is higher in these strains (*pmr1*Δ, *pmr1*Δ/*gdt1*Δ, and *pmr1*Δ* + GDT1*) when treated with FK506, an inhibitor of calcineurin (Fig. S4A).

### Gdt1p modulates total calcium content in the *pmr1*Δ mutant

To assess the effect of Gdt1p on the total Ca^2+^ content, we used inductively coupled plasma atomic emission spectroscopy (ICP-AES) to measure Ca^2+^ levels in the WT and the four mutants that we used in the aequorin-based assay. As shown in Fig. 2C, there was no significant difference in Ca^2+^ content between the WT and the *gdt1*Δ strain, whereas, in line with previous findings^21^, the whole-cell Ca^2+^ content of the *pmr1*Δ strain was significantly higher than that in the WT (10.6 versus 6.2 mmole/kg dry weight in WT cells, p < 0.05). In a *pmr1*Δ mutant, Ca^2+^ depletion of the secretory pathway occurs and is compensated by entry of calcium into the cell. As a result, the [Ca^2+^]_cyt_ increases, resulting in activation of the calcineurin signaling pathway, leading to overexpression of Pmc1p and, therefore, higher sequestration of Ca^2+^ into the vacuole^22^. Interestingly, as shown in Fig. 2C, the cellular Ca^2+^ concentration in the *pmr1*Δ mutant was dependent on *GDT1* expression, showing a decrease to 8.5 mmole/kg dry weight when *GDT1* was deleted and an increase to 13.2 mmole/kg dry weight when Gdt1p was overproduced. These results demonstrate that Gdt1p controls Ca^2+^ stores in yeast. As the yeast vacuole accumulates over 95% of the total cellular Ca^2+ ^23, the observed differences mainly reflect modifications of the vacuolar stock, and the variations observed in cellular Ca^2+^ content in *pmr1*Δ mutants expressing different levels of *GDT1* might explain the Ca^2+^ responses to osmotic shock as a result of modification of the amount of Ca^2+^ released through the vacuolar calcium channel Yvc1p.

### Gdt1p is required for protein glycosylation in yeast

Maintenance of a suitable intraluminal Ca^2+^ concentration is essential for the activity of many ER- and Golgi-resident enzymes involved in membrane trafficking and protein folding and glycosylation^8^,^9^. In this study, we examined whether Gdt1p was required for proper glycosylation of the vacuolar carboxypeptidase Y (CPY)^24^ and the glucanosyltransferase Gas1p^25^,^26^. CPY maturation, which involves N-linked glycosylation, is often used to study the efficiency of the secretory pathway. During its translocation through the ER and Golgi apparatus, CPY undergoes glycosylation at four sites, each glycan accounting for approximatively 2.5 kDa. Afterwards, the glycosylated precursor is delivered to the vacuole and the propeptide segment is proteolytically removed, generating the 61 kDa mature glycosylated form. Gas1p is also often used in glycosylation studies^27^ and undergoes both N-linked and O-linked glycosylation. A 105 kDa precursor is generated in the ER and is then processed in the Golgi apparatus to the 125 kDa mature glycosylated form. N-deglycosylation results in a 95 kDa protein and complete deglycosylation in a 58 kDa protein.

**Figure 3:**
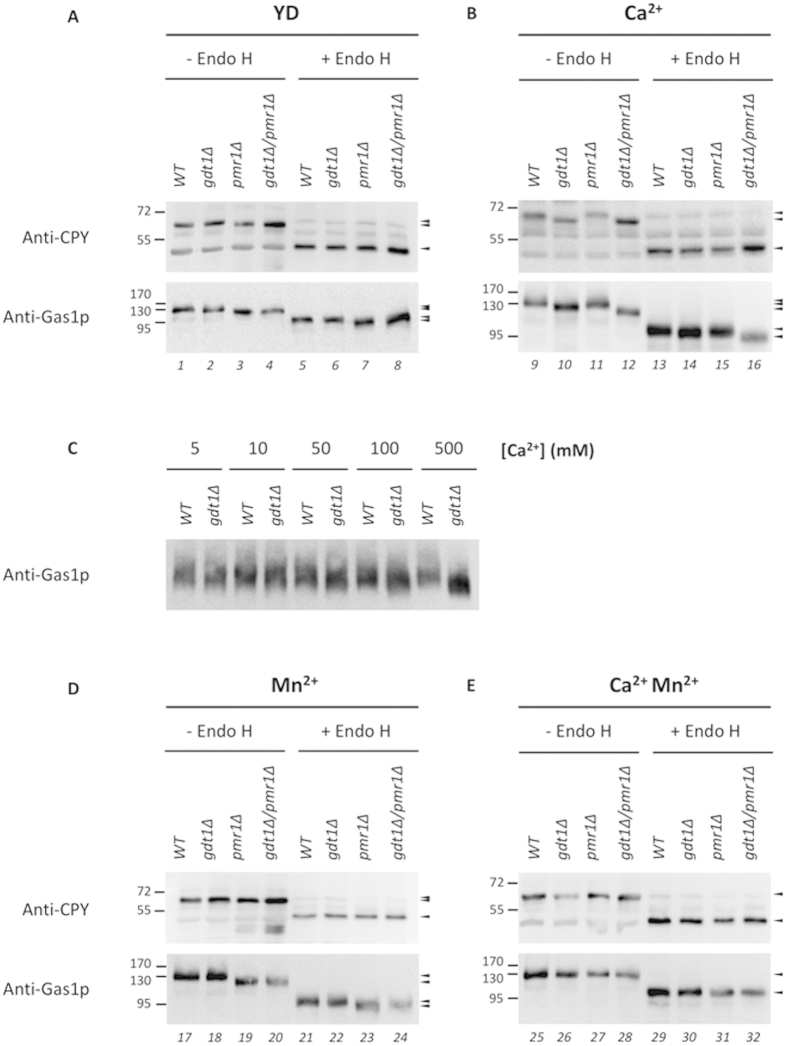
Glycosylation of CPY and Gas1p is impaired in the *gdt1*Δ mutant in the presence of 500 mM external Ca^2+^. Total membrane protein extracts of the indicated strains were prepared from cultures grown to an OD_600_ of 1.2 in YD medium (YD) alone **(A)** or supplemented with 500 mM CaCl_2_ (Ca^2+^) **(B)**, increasing Ca^2+^ concentrations **(C)**, 500 μM MnCl_2_ (Mn^2+^) **(D)** or both 500 mM CaCl_2_ and 500 μM MnCl_2_ (Ca^2+^Mn^2+^) **(E)**. Where indicated, the proteins were digested with endoglycosidase H (0.5 U/ml). Levels of CPY and Gas1p were then analyzed by SDS-PAGE followed by Western blotting analysis using specific antibodies. The different forms are indicated by arrows. All displayed results are representative of those seen in at least three replicates.

In order to assess the involvement of Gdt1p in protein glycosylation, we used Western blotting to examine glycosylation of CPY and Gas1p in the WT and mutants deleted for *GDT1* and/or *PMR1* grown either in YD medium alone or in YD medium containing 500 mM Ca^2+^. As shown in Fig. 3A, both CPY and Gas1p in lysates of the *pmr1*Δ mutant grown in YD medium migrated more rapidly on SDS gels (lane 3) than those in WT lysates (lane 1), but this difference was not seen when CaCl_2_ was added to the growth medium (lane 11) (Fig. 3B). This is in good agreement with previous studies on CPY reporting that the size difference results from a glycosylation defect in the Golgi apparatus^8^, and that addition of Ca^2+^ bypassed *pmr1*Δ glycosylation defects^28^. Interestingly, as shown in Fig. 3A,B, the opposite phenotype was observed for *gdt1*Δ mutants, as loss of *GDT1* did not seem to impair CPY and Gas1p maturation in YD medium (lane 2), but did in Ca^2+^-supplemented YD medium (lane 10). It is likely that these smaller mature forms reflect defects in glycosylation probably resulting from deregulation of the Ca^2+^ concentration in the Golgi caused by *GDT1* deletion and a high external Ca^2+^ concentration. As shown in Fig. 3C, this mobility shift was already visible in *gdt1*Δ cells grown in presence of smaller Ca^2+^ concentrations (50 mM) and appeared to increase when the Ca^2+^ concentration is rising. The size differences observed for the double deletant *gdt1*Δ*/pmr1*Δ arise from impaired glycosylation in YD medium due to the absence of *PMR1* (lane 4) and in Ca^2+^-containing medium due to the absence of *GDT1* (lane 12). Note that the mature form of Gas1p in the *gdt1*Δ*/pmr1*Δ mutant is even smaller (lane12), suggesting that both N- and O- glycosylations are impaired.

To analyze whether these mobility shifts were due to alterations in the N-glycosylation, the proteins were treated with endoglycosidase H (Endo H) before Western blotting. As shown in Fig. 3A,B, enzymatic removal of N-glycans from CPY led to one unique smaller size product within all four strains in both YD medium (lanes 5–8) and YD medium with added Ca^2+^ (lanes 13–16). These results confirm that N-linked glycosylation of CPY was impaired in *pmr1*Δ grown in YD medium and highlight the requirement for Gdt1p in order for N-glycosylation to take place in the presence of high calcium. As shown in Fig. 3A,B, slightly different results were obtained for Gas1p, which, after treatment with Endo H, showed a higher mobility in the *pmr1*Δ mutant than in the WT and *gdt1*Δ mutant in YD medium (lane 7) and in the *gdt1*Δ mutant than in the other two strains in Ca^2+^-supplemented YD medium (lane 14). Note that, depending on the growth medium, after Endo H digestion, the *gdt1*Δ*/pmr1*Δ strain showed a phenotype comparable to either the *pmr1*Δ strain (lane 8) or the *gdt1*Δ strain (lane 16). As Gas1p is subjected to both N-linked and O-linked glycosylation^25^ and Endo H exclusively cleaves N-linked glycans, we propose that these changes in Gas1p mobility reflect impaired O-linked glycosylation. The role of Pmr1p in O-glycosylation was already known^9^, but the involvement of Gdt1p in O-glycosylation in the presence of Ca^2+^ is a new observation.

Thus, our results demonstrate, for the first time, that Gdt1p is required for both N-linked and O-linked protein glycosylation at a high external calcium concentration and are consistent with the fact that mutation in the human ortholog, TMEM165, is linked to a genetic disease (Congenital Disorders of Glycosylation) that is caused by defects in glycosylation^16^.

### Mn^2+^ restores the glycosylation defect observed in *gdt1*Δ mutant

Mn^2+^ is an important cation required as cofactor for many glycosyltransferases and glycosidases involved in glycosylation^29^,^30^. We analyzed the glycosylation pattern of CPY and Gas1p in presence of 500 μM Mn^2+^ alone (Mn^2+^) or combined with 500 mM Ca^2+^ (Ca^2+^Mn^2+^). A similar glycosylation profile to that found in cells grown in YD medium was observed for CPY and Gas1p in presence of Mn^2+^ (Fig. 3D). Addition of Mn^2+^ in the growth medium did not affect the glycosylation in the wild type (lane 17) and *gdt1*Δ strain (lane 18) and did not restore N-linked and O-linked glycosylation defects in *pmr1*Δ (lane 19) and *gdt1*Δ*/pmr1*Δ (lane 20) since changes in mobility were still observable. Note that the mobility shift observed for CPY seems less important in presence of Mn^2+^ compared to YD suggesting a possible partial restoration of the N-glycosylation. Strikingly no glycosylation defect could be observed in Ca^2+^Mn^2+^ medium (Fig. 3E), highlighting that the addition of Mn^2+^ to Ca^2+^-containing medium restores the glycosylation defects of *gdt1*Δ (lane 26) and *gdt1*Δ*/pmr1*Δ (lane 28) strains. Interestingly, no mobility shift could be observed after removal of N-glycans by Endo H treatment (lanes 29–32), confirming that the addition of Mn^2+^ to Ca^2+^-containing medium restores both N- and O-glycosylation pathways for Gas1p in the *gdt1*Δ mutants.

## Discussion

We recently suggested the existence of a novel Golgi-localized Ca^2+^ transport system involving members of the highly-conserved UPF0016 family and showed that the yeast protein Gdt1 and its human ortholog, TMEM165, are functionally related and involved in Ca^2+^ and pH homeostasis^13^. Mutation in TMEM165 is known to cause a subtype of CDG, a group of rare diseases characterized by defects in glycosylation^16^. In this study, we provided direct evidence that Gdt1p mediates Ca^2+^ transport across membranes. When direct transport assays were carried out in *L. lactis*, Gdt1p-dependent Ca^2+^ influx through the plasma membrane was observed upon addition of calcium to the external medium (Fig. 1B,C). In addition, we also highlighted the pH-dependency of this Ca^2+^ transport, as calcium influx increased with an increase in the external pH (Fig. 1D). These results indicate that Gdt1p is a Ca^2+^ transporter and suggest that Gdt1p could act as a Ca^2+^/H^+^ exchanger. In these experiments, it was assumed that the cytosolic pH of *L. lactis* was not modified by the external pH. Indeed, the Kd of Fura-2 for Ca^2+^ depends on the pH and directly affects Ca^2+^ concentration determination. It is generally accepted that cytosolic pH may vary from 0.1 unit per unit of external pH variation^43^. Nevertheless the intracellular pH dependency should be formally measured in *L. lactis* cells incubated at pH7.0 and 8.0.

Gdt1p therefore constitutes a novel Ca^2+^ system in the yeast Golgi apparatus distinct from the well-studied Ca^2+^/Mn^2+^-ATPase, Pmr1p, the major calcium pump under normal growth conditions. Pmr1p plays a dual function in the cell. Firstly, given its localization, it is required to provide a suitable Ca^2+^ (and Mn^2+^) concentration in the organelles involved in the secretory pathway. Secondly, it is responsible for the detoxification of the cytosol when the external Ca^2+^ concentration rises. The *pmr1*Δ mutant shows a growth defect on medium containing a low (~ 3 μM) or high (~400 mM) Ca^2+^ concentration reflecting, respectively, Ca^2+^ starvation of the secretory pathway organelles or toxic accumulation of Ca^2+^ in the cytosol^9^,^11^. At the cellular level, *PMR1* deletion induces depletion of the secretory Ca^2+^ pools. Then the cell integrity-related mitogen-activated protein kinase Slt2p becomes activated and stimulates Ca^2+^ influx by activating the high-affinity Ca^2+^ uptake system (HACS) in order to refill Ca^2+^ stores in organelles involved in the secretory pathway^3^. Although the rate of Ca^2+^ influx is increased in a *pmr1*Δ mutant, the rate of Ca^2+^ efflux is unaffected, leading to an elevated [Ca^2+^]_cyt_^3^,^21^ and activation of the Ca^2+^/calcineurin-dependent pathway^3^,^20^. Calcineurin inhibits Ca^2+^ uptake via the HACS channel by a negative feedback mechanism involving direct dephosphorylation of Cch1p^31^, but induces *PMC1* expression via Crz1p^22^. Ca^2+^ uptake into the vacuole via Pmc1p is then increased, which results in higher cellular calcium stores^21^. Our data for the *pmr1*Δ mutant are consistent with those described in the literature. In the absence of *PMR1*, we observed an increase in the resting [Ca^2+^]_cyt_ (Fig. 2A), *PMC1* expression (Fig. 2B), and cellular calcium stores (Fig. 2C). We also found that the *pmr1*Δ mutant showed a higher Ca^2+^ response after saline or osmotic stress than the wild type. This may be due to the higher Ca^2+^ stores observed in this strain, which could be responsible for greater release of Ca^2+^ through the Yvc1p channel. Calcineurin activation is observed by the overexpression of *PMC1* in *pmr1*Δ, *pmr1*Δ/*gdt1*Δ, and *pmr1*Δ* + GDT1* strains. Moreover FK506 clearly increases steady-state [Ca^2+^]_cyt_ in those strains probably because of the absence of *PMC1* overexpression.

We also investigated the involvement of Gdt1p in Ca^2+^ homeostasis. We demonstrated that Gdt1p has an impact on the Ca^2+^ response following saline stress (Fig. 2A) or osmotic stress (Fig. S2) and on the internal Ca^2+^ stocks (Fig. 2C). When the Golgi Ca^2+^-ATPase Pmr1p was absent, overexpression of *GDT1* induced an increase of the Ca^2+^ response intensity and total Ca^2+^ store, while the opposite results were seen in the double deletant *gdt1*Δ*/pmr1*Δ. These results could be interpreted as either a direct or indirect role of Gdt1p. The simplest interpretation would be that Gdt1p transports Ca^2+^ out of the Golgi and directly participates in the Ca^2+^ response. However, the Ca^2+^ response results from the integration of different parameters, each of which is capable of modifying the shape of the curve. We tested the effect of two of these parameters, namely the Ca^2+^ stores and the abundance of other transporters. Our results showed that the Ca^2+^ response correlated with the total Ca^2+^ store level, which potentially influences the amount of Ca^2+^ released through Yvc1p, and that this correlation was dependent on Gdt1p. However, the mechanism by which *GDT1* overexpression increased the Ca^2+^ stores is still unknown. One possibility is that Gdt1p transports Ca^2+^ from the cytosol to the Golgi lumen and the Ca^2+^ then travels through the cell by vesicular trafficking and accumulates in the vacuole. In addition, we observed that Pmr1p, Pmc1p, and Yvc1p levels were not altered in the *gdt1*Δ mutant compared to the WT, whereas Vcx1p levels were modified in several strains tested (*pmr1*Δ, *pmr1*Δ/*gdt1*Δ, and *pmr1*Δ* + GDT1*) and an inverse correlation was found between Vcx1p levels and the Ca^2+^ response. This observation is compatible with the proposed role of Vcx1p in Ca^2+^ reabsorption after a transient increase in its cytosolic concentration^4^. However, Vcx1p activity is difficult to evaluate, since, in the *pmr1*Δ strains, the calcineurin pathway is activated and Vcx1p should be inhibited^6^.

Together, our data clearly demonstrate that Gdt1p plays an important role in Ca^2+^ homeostasis. One important question that requires answering is the direction of Ca^2+^ transport. From a thermodynamic point of view, both directions can be considered. In the hypothesis of a Ca^2+^/H^+^ exchange, and applying the Gibbs equation to the concentration values reported in literature, the transport of Ca^2+^ against its gradient (from the cytosol to the Golgi apparatus) would be thermodynamically feasible in exchange for 3 H^+^. On the other hand, Gdt1p could acidify the Golgi apparatus by transporting H^+^ against its gradient in a stoichiometry 1:1 (1 H^+^ for 1 Ca^2+^) or 2:1 (2 H^+^ for 1 Ca^2+^). To date, our current data do not provide the answer, but this point could be resolved, for instance, if an adapted Golgi-localized luminal calcium sensor could be engineered. Note that we cannot exclude the possibility that Gdt1p transport would be reversible, adapting the direction of transport according to conditions.

Using a complementation assay on Ca^2+^-containing medium, we previously demonstrated that the human ortholog TMEM165 is able to restore the growth defect observed in *gdt1*Δ on Ca^2+^-containing medium^13^, indicating that the function is conserved through evolution. Recently, Foulquier *et al*.^16^ reported that mutations of TMEM165 are involved in a subtype of CDG, inborn metabolic diseases linked to defects in the glycosylation pathway. Using MALDI-TOF analysis, they observed a slight defect in sialylation and galactosylation of N-glycans in TMEM165-deficient patients. In the present study, we showed that, in the presence of a high external Ca^2+^ concentration, Gdt1p was required for N-linked and O-linked protein glycosylation in yeast. Interestingly, Gdt1p and Pmr1p affected glycosylation in different ways, as we showed that CPY and Gas1p glycosylation defects occurred in the *pmr1*Δ strain in YD medium and were overcome by addition of Ca^2+^, whereas impaired glycosylation in the *gdt1*Δ strain was only observed in the presence of added Ca^2+^ (Fig. 3). Glycosylation requires a suitable concentration of both Ca^2+^ and Mn^2+^ in the ER and Golgi^9^. Mn^2+^ is needed as cofactor for various enzymes involved in the addition of carbohydrates to proteins undergoing N- and O-glycosylation^32^, while Ca^2+^ is important for membrane protein trafficking through the secretory pathway^33^. In this context, it was recently reported that addition of CaCl_2_ overcomes the glycosylation defect in the *pmr1*Δ mutant by stimulating intra-organelle redistribution through intracellular vesicle trafficking of Mn^2+^ imported into the ER via Spf1p and into the *trans-*Golgi apparatus via Smf2p^28^. In our study, we confirmed the important role of Ca^2+^ in Mn^2+^ redistribution as we showed that the addition of Mn^2+^ alone in the growth medium did not restore the glycosylation defects observed in *pmr1*Δ cells. In contrast to the results for the *pmr1*Δ strain, lack of Gdt1p only altered glycosylation in the presence of Ca^2+^, suggesting that the Ca^2+^ concentration in the Golgi lumen was increased. Based on these observations, Gdt1p would extrude Ca^2+^ from the Golgi to the cytosol. However, this is currently only a hypothesis as the direction of transport by Gdt1p is not yet known. In this context, restoration of glycosylation defects by Mn^2+^ in *gdt1*Δ (and *gdt1*Δ*/pmr1*Δ) may be explained by two ways. First, if we consider that Ca^2+^ concentration is too high in *gdt1*Δ mutant, Mn^2+^ could compete with Ca^2+^ to enter into the Golgi apparatus via the Mn^2+^/Ca^2+^-ATPase Pmr1p and therefore reduce the total intraluminal Ca^2+^ content. Alternatively, Ca^2+^ could compete with Mn^2+^ ions within the ER/Golgi lumen and alter glycosylation process. In this case, adding back more Mn^2+^ could restore the defect. Measuring the ions concentration in the organelle lumen would help to answer this question.

In conclusion, we have demonstrated that Gdt1p is a calcium transporter localized in the Golgi apparatus and plays a crucial role in calcium homeostasis and protein glycosylation. Our results provide new insights into the molecular causes of the defect in glycosylation described in TMEM165-deficient patients.

## Experimental procedures

### Strains, culture media, and growth conditions

The *Saccharomyces cerevisiae* strains used are listed in Table 1. The BY4741 or BY4742 background strains were purchased from the Euroscarf systematic deletion library (kanamycin deletion cassette). The double-deletant created in this study was obtained by crossing the two single deletants. Non-transformed yeast cells were routinely cultured at 28 °C in YD medium (2% yeast extract KAT, 2% glucose). Cells transformed with plasmids were grown in SD minimal medium [0.7% yeast nitrogen base without amino acids (Difco), 2% glucose, supplemented with all amino acids except those used as selection markers for plasmid maintenance]. Solid media were produced by addition of 2% agar to the mixture. Where indicated, calcium chloride was added at the required concentration; the required amount of calcium chloride dissolved in 50 ml of distilled water was autoclaved and added to the autoclaved medium to avoid precipitation. *Lactococcus lactis* NZ9000 wild type strain and its derivative, the evolved DML1 strain, were kindly provided by B. Poolman (Groningen, Holland)^19^; strains transformed with pNZ8048-10His-strep-TEV-Δ23GDT1 (See below) were grown in M17 medium (Merck) supplemented with 1% glucose and 10 μg/ml of chloramphenicol at 28 °C without agitation. After preliminary trials to determine the optimal nisin concentration and induction time for the highest Gdt1p expression (Fig. S1), expression was induced under the control of the nisA promoter by adding nisin at a final concentration of 2.5 μg/L to cultures in the log phase (OD_600_ ~0.4–0.5) and harvesting the cells 3 hours later.

### Vector construction

Yeast and bacterial plasmids were obtained following standard molecular biology protocols, and the authenticity of all genetic constructs was validated by sequencing. pRS416-pTPI-GDT1, the yeast plasmid overexpressing *GDT1*, was obtained previously and has been described by Demaegd *et al*.^13^. Yeast transformation was performed following the method of Gietz *et al*.^34^. For the heterologous expression of Gdt1p in *L. lactis*, we used the pNZ8048 plasmid expressing a tagged version of *GDT1* lacking the 23 first amino acids corresponding to the predicted signal peptide (pNZ8048-10His-strep-TEV-^Δ23^*GDT1*) under the control of the nisin-inducible promoter. This plasmid was constructed as follows. The yeast pRS416 vector containing the sequence coding for the 10His-strep-TEV tagged ^Δ23^*GDT1* was used as the DNA template for PCR amplification, then the amplified PCR products were digested with Pst1/SacI and inserted into the pNZ8048 vector carrying the nisin-inducible promoter. Bacterial transformations were performed by electrotransformation (Bio-Rad Laboratories) as described previously by Holo *et al*.^35^ and transformants selected by chloramphenicol resistance.

### Preparation of the total membrane fraction from *L. lactis*

Recombinant Gdt1p proteins were expressed in *L. lactis* as described above, then the cells were harvested (1,700 g for 12 min at 4 °C) and washed once with washing buffer (50 mM Tris/HCl pH 7.6, 500 mM NaCl, and 10% glycerol), and centrifuged as above, then the pellet was resuspended in one volume of ice-cold lysis buffer [50 mM Tris/HCl pH 7.6, 500 mM NaCl, 10% glycerol, 1 mM Tris(2-carboxyethyl) phosphine (TCEP), 1 mM PMSF, 2 mg/ml lysozyme, and protease inhibitor cocktail (PIC, 4 μg/ml of leupeptin, aprotinin, antipain, pepstatin, and chymostatin). After 30 min incubation at 28 °C, the cells were lysed with glass beads (cell pellet/ice-cold lysis buffer/glass beads at a weight ratio of 1:1:1) using a Precellys cell disrupter (Bertin Technologies) at 5,000 RPM for 5 × 30 sec. Cells debris were removed by centrifugation at 1,700 g for 12 min at 4 °C and the supernatant centrifuged at 112,000 g for 60 min at 4 °C to pellet the total membrane fraction, which was resuspended in ice-cold resuspension buffer (50 mM Tris/HCl pH 7.6, 500 mM NaCl, 10% glycerol, 1 mM TCEP, 1 mM PMSF, and PIC) and stored at −80 °C.

### Yeast crude membrane extracts

Yeast total membrane extracts were prepared from 100 ml of culture at an OD_600_ of 1.2 as described previously by Morsomme *et al*.^36^, except that dithiothreitol was not added at any step of the protocol and the last centrifugation was performed at a higher speed (100,000 g). The protein concentration was determined by the method of Smith *et al*.^37^.

### Antibodies and Western blotting

Routinely, 15–20 μg of membrane proteins was mixed with 4× concentrated non-reducing sample loading buffer (0.32 M Tris-HCl pH 6.8, 8% SDS, 40% glycerol, 0.02% bromophenol blue). Yeast samples were used as such, while *L. lactis* samples were incubated at 37 °C for 30 min. Membrane proteins were separated on a 10% SDS/PAGE gel and Western blotting performed as described previously^13^. Primary rabbit polyclonal antibodies against Pmc1p (1:125 dilution), Pmr1p (1:125 dilution), Yvc1p (1:500 dilution), or Vcx1p (1:500 dilution) were produced for this study by Perbio Science and were raised against a synthetic peptide designed specifically for each protein (Pmc1p, residues 1,155–1,173; Pmr1p, residues 932–950; Yvc1p, residues 657–675; Vcx1p, residues 13–26 + 399–411). The other primary antibodies used were rabbit anti-Gdt1p [1:333; produced previously in our laboratory^13^], rabbit anti-Gas1p (1:2,000; gift from H. Riezman, Geneva, Switzerland), and rabbit anti-CPY (1:2,000; gift from H. Riezman, Geneva, Switzerland). Horseradish peroxidase-coupled anti-rabbit IgG antibodies (1:10,000 dilution) were purchased from IMEX.

### Endoglycosidase H digestion

Samples (17 μg) of total membrane protein were precipitated using chloroform/methanol as described by Wessel *et al*.^38^, then solubilized by boiling for 10 min in 16 μl of denaturation buffer [50 mM sodium citrate, pH 5.5 (HCl), 0.5% (w/v) SDS, 0.1 M β-mercaptoethanol], followed by addition of 1 mM PMSF, PIC, and 23 μl of citrate buffer [50 mM sodium citrate, pH 5.5 (HCl)] either alone or containing 0.5 unit/ml of endoglycosidase H (Roche). After incubation for 30 min at 30 °C, the reaction was stopped by addition of 15 μl of 4× sample loading buffer.

### *In vivo* Ca^2+^ transport assay in *L. lactis* cells

Intracellular Ca^2+^ concentrations in *L. lactis* were measured using the fluorescent calcium dye Fura-2/AM, following the method previously described by Chang *et al*.^39^, with minor modifications: these were a longer incubation time of cells in the presence of EDTA (30 min at 30 °C without shaking), addition of 1.7 mM probenecid to solution A in the Fura-2 loading step in order to limit its leakage^40^, and addition of 0.1 mM EGTA to solution A prior to measurement. To assess the pH dependency of calcium transport, the Fura-2/AM-loaded *L. lactis* cells were resuspended in solution A containing a final concentration of 0.1 mM EGTA and 50 mM Tris-HCl (final pH 7.0 or 8.0) and fluorescence measurements performed on 2 ml aliquots at 25 °C with constant stirring. The intracellular Ca^2+^ concentration was monitored as the change in the ratio of the fluorescence intensities (510 nm) at the excitation wavelengths of 340 nm and 380 nm using a JASCO FP8500 fluorimeter controlled by Spectra Manager software^TM^. The baseline fluorescence was routinely recorded every 10 sec for 2 min before addition of the indicated concentration of Ca^2+^. The fluorescence intensity ratio was converted into the Ca^2+^ concentration using the equation described by Liao *et al*. (K_d_ = 315 nM)^41^.

### Aequorin assay

Aequorin-based experiments were performed as described by Demaegd *et al*.^13^. In this case, we applied osmotic stress to a culture at an OD_600_ of 1.2 by adding a final concentration of 1.33 M NaCl or 2.66 M. sorbitol.

### Measurement of whole-cell Ca^2+^ content

Yeast cultures were grown in 50 ml of synthetic medium at 28 °C to a final OD_600_ of 3, then were collected by vacuum filtration using membrane filters (Millipore, 0.45 μm pore size) and washed successively with 1 mM ethylene glycol tetraacetic acid disodium salt solution and water. The cells were then resuspended in 10 ml of water and dried in an oven at 70 °C for 24 h, then in a desiccator for 24 h. The dried matter was weighted and mineralized by heating at 500 °C overnight, then the ashed sample was dissolved in 65% HNO_3_ and used for inductively couple plasma atomic emission spectroscopy (ICP-AES) analysis. Ca^2+^ measurements were performed using a ICAP 6500 spectrometer (Thermo Scientific) and the cellular Ca^2+^ concentration calculated based on the dry weight of the samples and the dilution factor.

## Additional Information

**How to cite this article**: Colinet, A.-S. *et al*. Yeast Gdt1 is a Golgi-localized calcium transporter required for stress-induced calcium signaling and protein glycosylation. *Sci. Rep.* **6**, 24282; doi: 10.1038/srep24282 (2016).

## Supplementary Material

Supplementary Information

## Figures and Tables

**Table 1:** Yeast strains and mutants used in this study.

Strain	Genotype	Source
BY4741	*Mata his3*Δ*1 leu2*Δ*0 met15*Δ*0 ura3*Δ*0*	Euroscarf
BY4741 *gdt1*Δ	*Mata his3*Δ*1 leu2*Δ*0 met15*Δ*0 ura3*Δ*0 gdt1::KanMX4*	Euroscarf
BY4742 *pmc1*	*Matα his3*Δ*1 leu2*Δ*0 lys2*Δ*0 ura3*Δ*0 pmc1::KanMX4*	Euroscarf
BY4741 *pmr1*Δ	*Mata his3*Δ*1 leu2*Δ*0 met15*Δ*0 ura3*Δ*0 pmr1::KanMX4*	Euroscarf
BY4741 *vcx1*Δ	*Mata his3*Δ*1 leu2*Δ*0 met15*Δ*0 ura3*Δ*0 vcx1::KanMX4*	Euroscarf
BY4741 *yvc1*Δ	*Mata his3*Δ*1 leu2*Δ*0 met15*Δ*0 ura3*Δ*0 yvc1::KanMX4*	Euroscarf
BY *gdt1*Δ*/pmr1*Δ	*Mata his3*Δ*1 leu2*Δ*0 ura3*Δ*0 lys2*Δ*0 met15*Δ*0 gdt1::KanMX4 pmr1::KanMX4*	13
