# Supplementary material for: Yeast Gdt1 is a Golgi-localized calcium transporter required for stress-induced calcium signaling and protein glycosylation
Source: Sci Rep. 2016 Apr 14;6:24282. doi: 10.1038/srep24282 (PMC4830978; doi:10.1038/srep24282)
Supplement: Supplementary Information [file srep24282-s1.pdf]

Supplementary data

Yeast Gdt1 is a Golgi-localized calcium transporter required for stress-induced calcium signaling and protein glycosylation

Anne-Sophie Colinet[1,3], Palanivelu Sengottaiyan[1,3], Antoine Deschamps[1], Marie-Lise Colsoul[1], Louise Thines[1], Didier Demaegd[1], Marie-Clémence Duchêne[1], François Foulquier[2], Pascal Hols[1], Pierre Morsomme[1*]

[1]Institut des Sciences de la Vie, Université catholique de Louvain, B-1348 Louvain-la-Neuve, Belgium
[2]UMR8576 CNRS, Structural and Functional Glycobiology Unit, University of Lille 1, IFR 114, F-59655 Villeneuve D'Ascq, France
[*]To whom correspondence should be addressed. E-mail: pierre.morsomme@uclouvain.be
[3]A.-S.C. and P.S. contributed equally to this work.

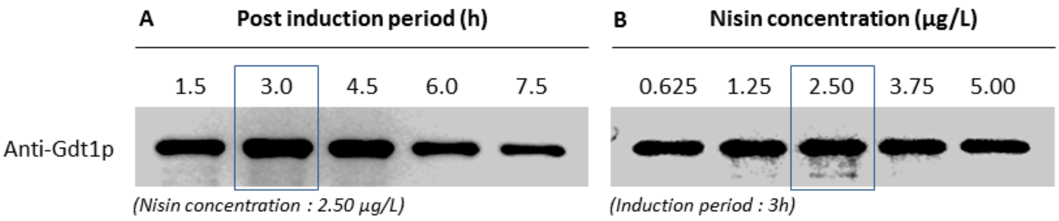

Figure S1. Addition of 2.5 µg/L nisin for 3 hours gives optimal induction of *GDT1* expression in *L. lactis*. *L. lactis* NZ9000 wild type cells expressing 10His-Strep-TEV-$^{\Delta 23}$GDT1 were incubated **(A)** for 1.5-7.5 h with 2.5 µg/L of nisin A or **(B)** for 3 h with 0.625-5.0 µg/L of nisin A, then GDT1 levels were assessed on total membrane protein fractions by SDS-PAGE, followed by Western blotting with anti-Gdt1p antibodies.

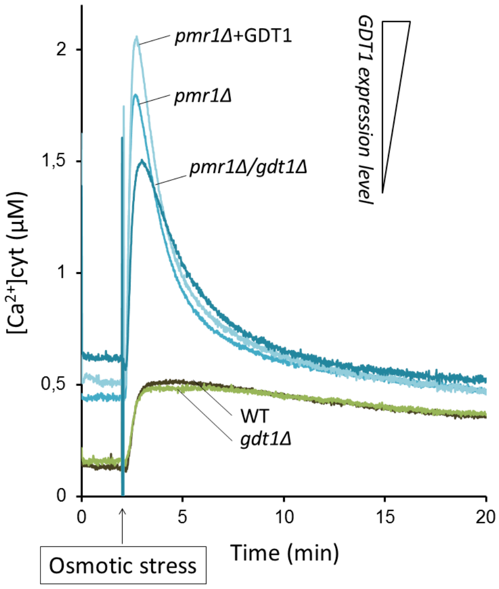

Figure S2. Gdt1p is involved in the calcium response in a *pmr1Δ* mutant after sorbitol shock. The experiment was identical to that in Fig. 2A, except that 2.66 M sorbitol (osmotic stress) was used instead of NaCl.

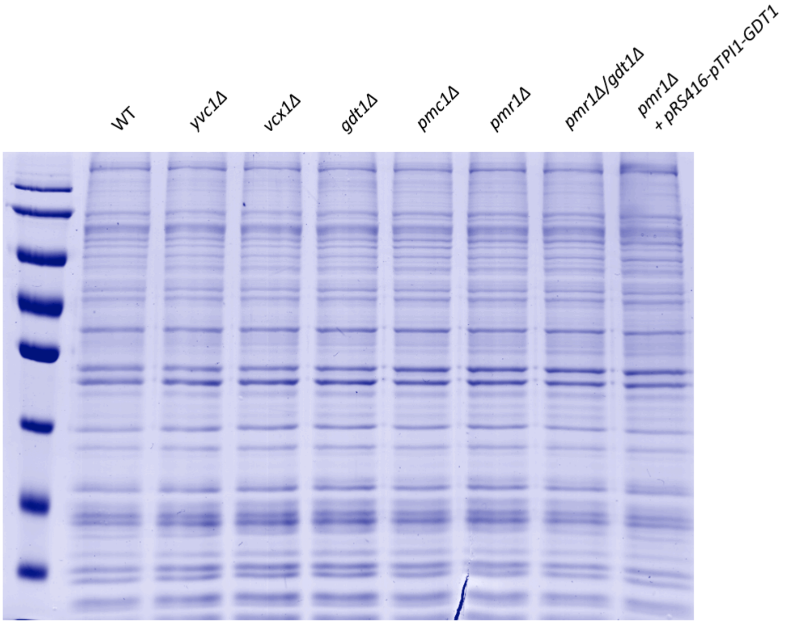

Figure S3. Coomassie blue-stained SDS-PAGE gel used as the loading control for Figure 2.

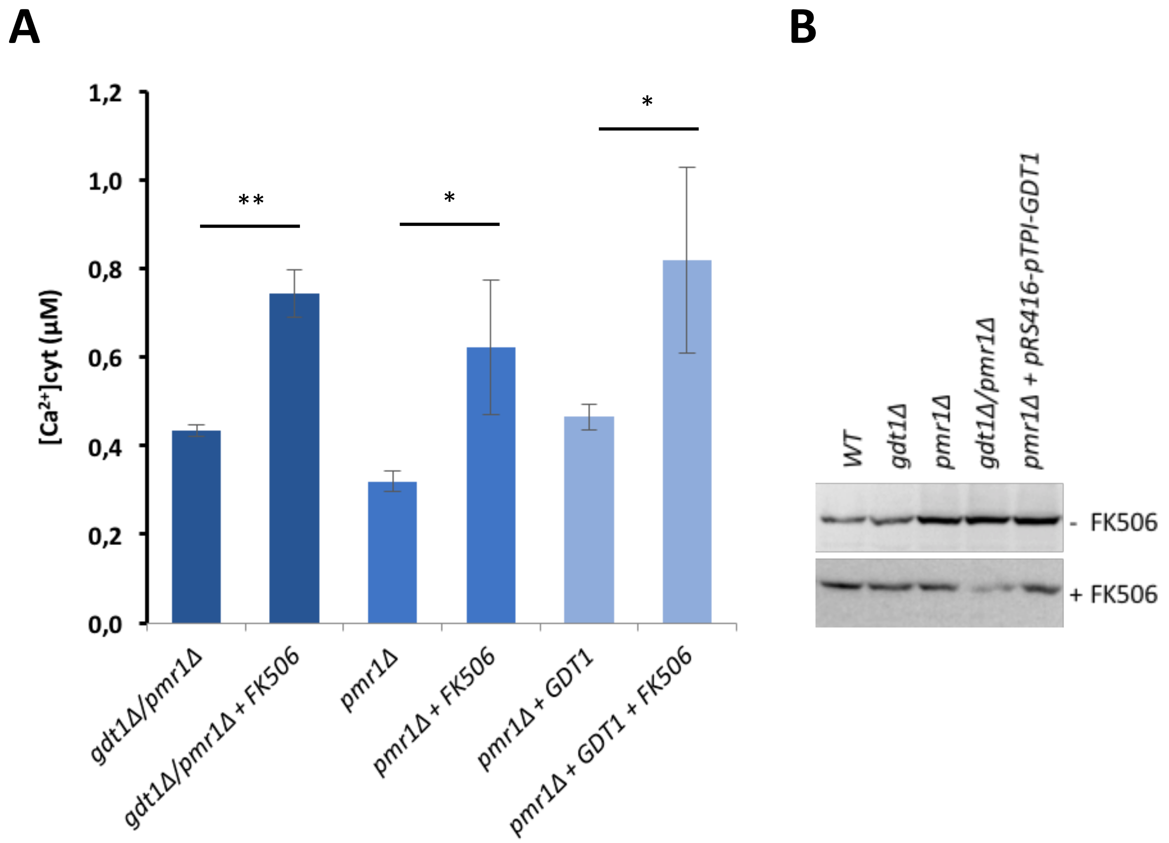

Figure S4. The calcineurin is activated in the *pmr1Δ*, *pmr1Δ/gdt1Δ* and *pmr1Δ* + GDT1. The experiment was identical to that in Fig. 2A and the cytosolic calcium concentration was measured at basal state. Cells treated with FK506 were grown overnight with 0.4 µg/ml of the drug. The data were analyzed by a Student's t-test. The values are expressed as the mean ± S.E.M (n=3), * : P < 0,05 ; ** : P < 0.01.

Table S1. Cell density of the Fura-2 loaded *L. lactis* cells expressing tagged-$^{\Delta23}$Gdt1p in buffers at different pHs before and after addition of 0.5 mM $CaCl_2$. Results are expressed as the mean ± S.D. for three separate experiments.

Assay buffer pH	OD_{600} before calcium addition	OD_{600} after calcium addition
7	1.52 ± 0.25	1.41 ± 0.18
8	1.54 ± 0.23	1.45 ± 0.23
